# Preliminary Experience With the New Amplatzer™ Trevisio™ Delivery System in Transcatheter Atrial Septal Defect Closures in Children

**DOI:** 10.3389/fped.2021.641742

**Published:** 2021-03-11

**Authors:** Raymond N. Haddad, Diala Khraiche, Damien Bonnet, Mathilde Meot, Sophie Malekzadeh-Milani

**Affiliations:** ^1^M3C-Necker, Hôpital Universitaire Necker-Enfants Malades, Assistance Publique-Hôpitaux de Paris (AP-HP), Paris, France; ^2^Université de Paris, Paris, France

**Keywords:** Amplatzer™ Trevisio™ intravascular delivery system, amplatzer, device closure, cardiac catheterization, atrial septal defect

## Abstract

**Objectives:** To evaluate safety, efficacy, and technical advantages of Amplatzer™ Trevisio™ intravascular delivery system (ATIDS) in percutaneous atrial septal defect (ASD) closure in children.

**Background:** The Trevisio™ is a novel delivery system designed for accurate and facilitated implantation of Amplatzer™ devices. There are no published clinical reports so far.

**Methods:** During September 2020, 9 children with anatomically challenging ASDs underwent attempted transcatheter closure using ATIDS to deliver Amplatzer™ Septal occluders (ASO). All interventions were performed under general anesthesia, trans-esophageal echocardiography (TOE), and fluoroscopic guidance. Standard safety, immediate, and 60-days outcomes were prospectively assessed.

**Results:** The median age was 8.1 (5.1–16.9) years and the median bodyweight was 30 (18–63) kg. Six patients had isolated secundum-type ASDs with absent anterosuperior rims including one with an aneurysmal septum. Three patients had unclassical defects associated with complex congenital heart anomalies. Eight devices were delivered from the femoral vein and the jugular vein was accessed in one patient with interrupted inferior caval vein and azygos continuation. All implantations were successful. The shape, position, and orientation of the ASO were identical before and after release on TOE and fluoroscopy. There was no device embolization or serious complication following closure. Complete shunt closure was confirmed on follow-up.

**Conclusions:** We report the first clinical experience with ATIDS in transcatheter ASD pediatric closures. Safety and efficacy were witnessed in our case-series. The major advantage of reduced-tension deployment and reliable precision in device positioning is highly beneficial in challenging anatomies and unusual access.

## Introduction

Since the first report of atrial septal defect (ASD) non-surgical closure in 1974, multiple occluders were tested in clinical studies (1–3). However, it was not until the Amplatzer™ Septal Occluder (ASO) became available in the mid-1990s that transcatheter ASD closure became a routine procedure (4). Today, the ASO remains the most widely used device with proven long-term efficacy and safety in adult and pediatric patients (5, 6). In some cases, implantation success may be limited by complex ASD anatomies with insufficient surrounding rims or limited venous access. New Amplatzer-like self-centering devices with modified delivery systems were developed by several manufacturers to overcome these difficulties but their behavior during implantation, and their medium-to-long-term performance is a subject of current debates (7–10). The ability of interventionists to tackle anatomically challenging defects has also progressed over time with substantial modifications in delivery techniques but was associated with increased procedural time, irradiation, and risk of complications (11, 12). The recently introduced Amplatzer™ Trevisio™ intravascular delivery system (ATIDS) for Amplatzer™ devices has an ultra-flexible delivery wire tip with promising technical advantages in terms of reduced-tension deployment and precise implantation. We report the first experience with the Trevisio™ delivery system in percutaneous ASD pediatric closures.

## Materials and Methods

### Study Design and Patients Selection

During September 2020, all consecutive pediatric patients with anatomically challenging ASDs and scheduled for attempted transcatheter closure using the new ATIDS to deliver Amplatzer™ Septal occluders (ASO) (AGA Medical Corp., MN, USA) at our institution were enrolled in this study and prospectively followed-up for 60 days. Written informed consent was signed by the patients' legal guardians to perform the procedure after they were provided with a comprehensive explanation about the procedural details. Permission was obtained from the manufacturer to use and mention their product in this submission. Standard safety, immediate and 1-month outcomes were assessed.

### Catheter Procedure

All procedures were performed in the catheterization laboratory by an experienced pediatric interventional cardiologist under general anesthesia, fluoroscopic control, and transoesophageal echocardiography (TOE) guidance. Before the procedure, all patients underwent detailed protocol transthoracic echocardiography (TTE) assessment by an experienced pediatric operator to evaluate the physiology, to delineate the anatomy, defect margins, and the presence of associated anomalies, and to confirm the indication of closure (13, 14). At the start of the procedure, the same echocardiographer performed careful TOE evaluation. The ASD sizing was based on strict 2-dimensional TOE measurements in 3 different planes (0°, 35° to 55°, and 90° to 110° angles) without static balloon sizing technique. As per-institutional protocol, the size of the device was selected based on the largest diameter (3–4 mm larger in defects with adequate anterosuperior rim and 5–6 mm larger in defects with absent anterosuperior rims). The right femoral vein was routinely accessed using a 7-Fr short introducer and in case of anatomic limitations, the approach was transjugular. Intravenous cefazolin and heparin were given according to protocol. A 0.035-inch wire was placed in the left or right upper pulmonary vein through the ASD using a 5-Fr multipurpose or Judkins right diagnostic catheter (Cordis Corporation, FL, USA). In complex cases, the wire was positioned in the left ventricular outflow tract. All ASO were delivered using ATIDS and deployed under continuous fluoroscopic and TOE guidance. The device was released, once the correct position and absence of residual shunt and interference with other structures were confirmed by TOE. Standard potential adverse events were assessed. The procedure was considered successful only when the device was released into the desired position with no residual shunt or immediate device embolization. Procedural time and irradiation data were recorded. All patients received standard care of the venous access site. They were followed up at 1, 7, 30, and 60 days by clinical examination, electrocardiogram, and TTE with a focus on device position, the competence of the atrioventricular valves, and the absence of pericardial effusion. Aspirin therapy was prescribed for 6 months.

### Amplatzer™ Trevisio™ Intravascular Delivery System

**Figure 1:**
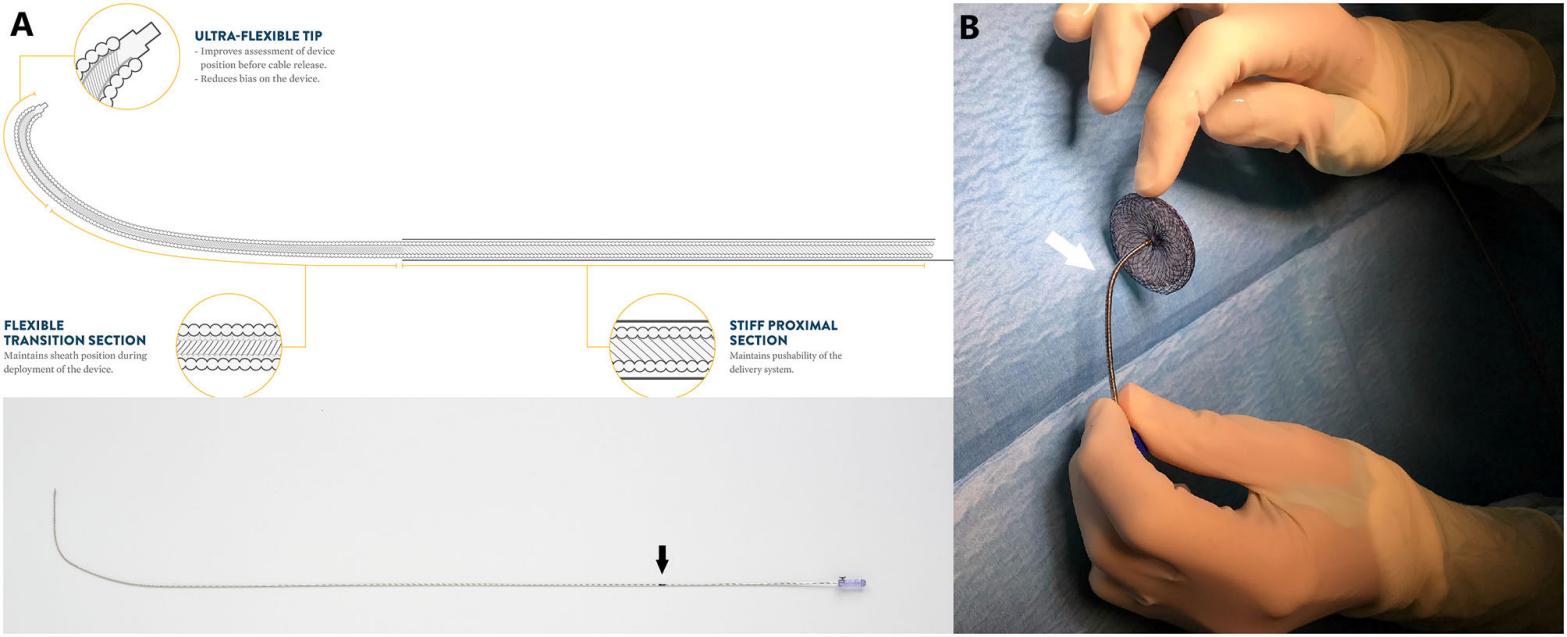
**(A)** Detailed schematic presentation of Amplatzer™ Trevisio™ intravascular delivery system with recently added features. Note the black marking on the proximal tip of the cable (Black arrow). **(B)** Flexible distal section (white arrow) of the Trevisio™ delivery wire connected to a 20 mm Amplatzer™ Septal occluder and manually bent to a 90° angle. Amplatzer and Trevisio are trademarks of Abbott or its related companies. Reproduced with permission of Abbott, ^©^2020. All rights reserved.

The ATIDS was recently introduced for the Amplatzer™ device family with promising enhanced deliverability. It leverages the one-piece cable design utilized by the Amplatzer™ TorqVue™ Delivery System, also known as the Classic Amplatzer™ Delivery System. The cable is designed with a stiff proximal section for no compromise on the torque strength, sheath diameter, and pushability. The flexible transition section stabilizes the position of the sheath during device deployment. The highly flexible wire-tip with the ultra-short screw reduces the bias on the device enhancing the assessment of device position before cable release (Figure 1A). These new flexibility features allow the distal part of the cable to easily tilt for more than a 90° angle optimizing the alignment of the device with the atrial septum during implantations (Figure 1B). The delivery system is shipped separately from the device with a 45° angle curvature sheath that is available in two lengths (60 or 80 cm). The ATIDS sheath size is exactly the same as used before, ranging from 6-Fr to 12-Fr. The body of the sheath is radiopaque for visibility under fluoroscopy. An ~0.25-inch long black mark is precisely positioned at 75.2 and 94.5 cm from the distal tip of the 110 cm- and 120 cm-long-cables in the 60 and 80 cm delivery systems, respectively (Figure 1A). It is added to inform the operator that when the marking reaches the Tuohy valve, the device is near the tip of the sheath.

### Statistical Analysis

Categorical variables were reported as frequency and percentage and continuous variables were represented as median with total range as appropriate.

## Results

**Figure 2:**
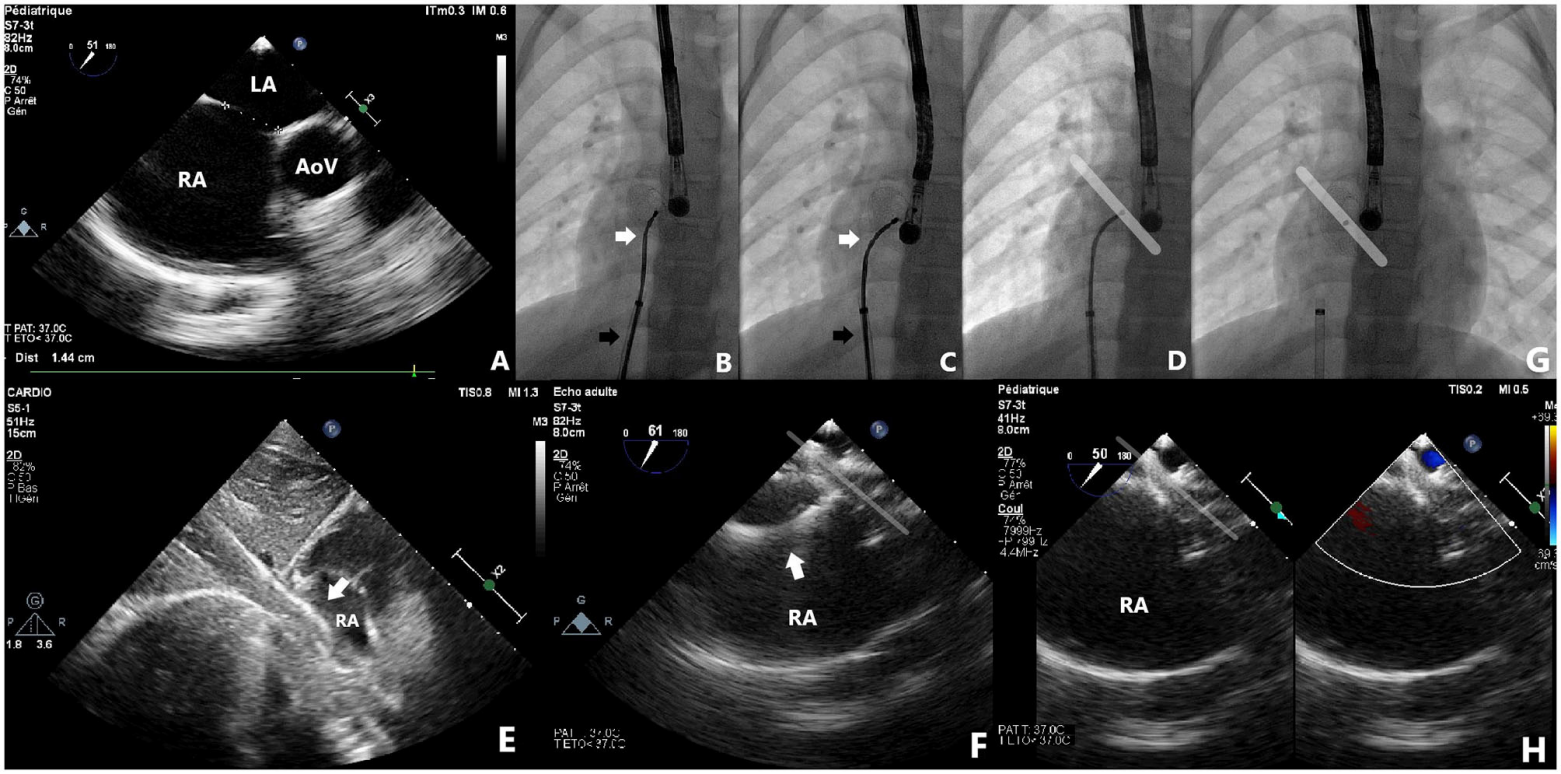
Case 9 presentation. Pre-procedural 2-dimensional and color Doppler TOE showing a large ASD with deficient aortic margin and dilated RA **(A)**. Note how the cable adapt (black arrows) and position the deployed device into its final definitive position once the sheath is rotated clockwise and the cable is gently pushed forward **(B–D)**. The flexible tip of the Trevisio™ delivery wire is clearly visualized on fluoroscopy **(D)**, TTE **(E)** and TOE **(F)**, resulting in less tension on the device and more favorable device orientation with identical post-release device position and orientation **(G,H)**. AoV, aortic valve; LA, left atrium, RA, right atrium.

**Figure 3:**
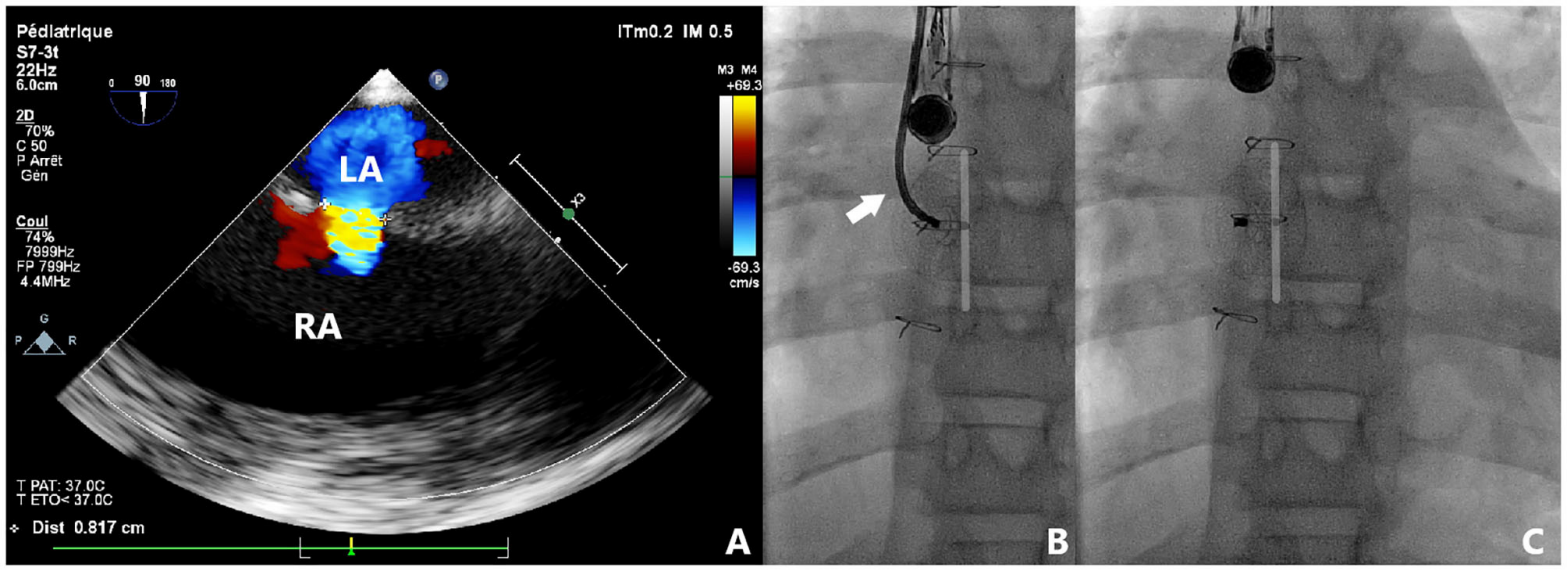
Case 4 presentation. Pre-procedural 2-dimensional and color Doppler TOE showing a centrally located and bidirectionally shunting ASD with all adequate rims **(A)**. Fluoroscopic views of transjugular device deployment using TrevisioTM system **(B)**. The flexible tip of the cable is visualized (white arrow) resulting in reduced tension and high accuracy in device positioning. Note the identical position, shape, and orientation of the device before **(B)** and after **(C)** release. LA, left atrium; RA, right atrium.

**Figure 4:**
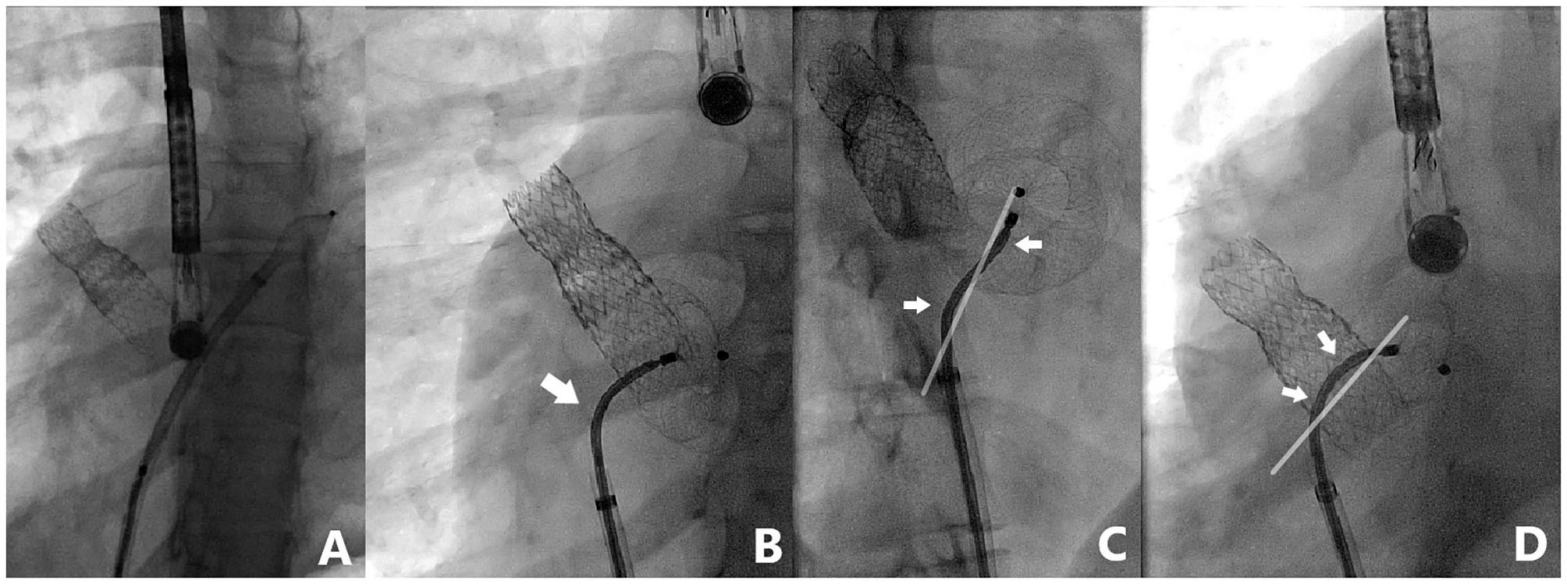
Case 5 presentation. Fluoroscopic views of a complex ASD closure case with previously stented right upper pulmonary vein. Note the highly performing 3-dimensional flexibility (white arrows) of TrevisioTM delivery wire allowing a wide range of angulation (up to 120° angle) with 2 inflection points for controlled and precise device deployment **(A–D)**.

Nine patients were included and their clinical characteristics are outlined in Table 1. The median age was 8.1 years (ranging from 5.1 to 16.9 years). Six patients had isolated secundum-type ASD with inadequate anterosuperior rim and heart dilatation (Figure 2A) including one with aneurysmal interatrial septum. One patient with surgically-corrected double outlet right ventricle and straddling mitral valve had left-to-right shunt across a residual ASD initially created by balloon atrial septostomy in the neonatal period and left untreated until the age of 5 years. Another patient with left isomerism, interrupted inferior caval vein, azygos continuation, surgically-repaired complete atrioventricular septal defect with 2 subsequent surgeries for regurgitation and stenosis of the right atrioventricular valve presented with exercise-induced oxygen desaturation and chronic migraines. She was diagnosed with bidirectional shunt across the surgically created-ASD that was initially left for residual mild tricuspid stenosis (Figure 3A). Another patient with a medical history of extreme prematurity, severe form of pulmonary bronchodysplasia, and large secundum-type ASD progressively developed pulmonary hypertension of mixed origin at the age of 2 years. She had aggressive medical treatment with satisfactory outcomes. Right superior pulmonary vein stenosis was diagnosed after 4 years and was treated with balloon dilation and repetitive stenting (Figure 4A). At the age of 17 years, the mean pulmonary pressures dropped to 25 mmHg and the left-to-right shunt across the ASD was significant (Qp/Qs = 1.8) with an indication of closure. Devices were delivered from the right femoral vein in 8 patients and the right jugular vein in one patient. Median procedural and fluoroscopic times were, respectively, 16 min (range, 8–40 min) and 4.9 min (range, 1.04–17.2 min). All devices were accurately and uneventfully delivered, positioned, deployed, into proper position. Device release was controlled and successful in all cases without any technical difficulty. No major complications occurred in any of the subjects. The 60-days follow-up confirmed complete shunt closure.

**Table 1:** Patients' clinical characteristics.

**Case**	**Gender**	**Age (years)**	**W (Kg)/H (cm)**	**CHD**	**ASD size (mm)**			**Aortic rim**	**Venous access**	**Guide position**	**ASO size (mm)**	**Sheath size (Fr)**	**PT (min)**	**FT (min)**	**K_**ar**_ (mGy)/DAP (μGym^**2**^)**	**Complex procedure**
					**0°**	**SOV**	**BCV**									
1	Male	5.6	21/122	ASD OS	15.8	17	17.3	Absent	Femoral	RUPV	22	9	25	2.8	3.6/58	No
2	Female	9.8	43/140	ASD OS	21	18.8	21.2	Absent	Femoral	LUPV	26	10	40	10.9	30.1/602.4	Yes
3	Male	5.1	18/110	DORV—SMVCorrective surgeryPost-BAS residual ASD	14.2	12.9	15.1	Adequate	Femoral	LVOT	18	8	38	17.2	51.3/380.2	Yes
4	Female	9.9	39/150	LI—CAVC—TSICorrective surgery—TPSurgically created residual ASD	5.9	6	8.1	Adequate	Jugular	LVOT	12	7	10	4.9	7.7/77.5	Yes
5	Female	16.9	63/160	PHTN—RUPV stenosisRUPV Ballooning + stentingASD OS	16.6	14.5	16.3	Absent	Femoral	LUPV	22	9	24	6.1	96/916.8	Yes
6	Female	6.6	20/117	ASD OS	16.1	16	16.3	Absent	Femoral	RUPV	22	9	16	9.12	4.6/93.9	No
7	Female	13.4	49/167	ASD OSAneurysmal inter-atrial septum	12.9	12	13	2mm	Femoral	LUPV	18	8	16	3.92	2.3/39.4	No
8	Male	8.1	30/127	ASD OS	13.8	11.4	12.8	Absent	Femoral	RUPV	18	8	8	1.04	2/22.6	No
9	Female	7.6	25/130	ASD OS	16.6	14.4	14	Absent	Femoral	LUPV	22	9	10	1.5	1.3/22.7	No
Median	–	8.1	30/130	–	15.8	14.4	15.1	–	–	–	22	9	16	4.9	4.6/77.5	–

*W/H, weight/height; CHD, congenital heart disease; ASD, atrial septal defect; SOV, short axis view; BCV, bi-caval view; ASO, Amplatzer septal Occluder; PT, procedural time; FT, fluoroscopic time; K_ar_, cumulative air kerma at the patient entrance reference point; DAP, dose area product; OS, ostium secundum; DORV, double outlet right ventricle; SMV, straddling mitral valve; BAS, balloon atrial septostomy; LI, left isomerism; CAVC, complete atrioventricular canal; TSI, tricuspid stenosis and insufficiency; TP, tricuspid plasty; PHTN, pulmonary hypertension; RUPV, right upper pulmonary vein; LUPV, left upper pulmonary vein; LVOT, left ventricular outflow tract*.

## Discussion

The ASO continues to be the most widely used device for ASD closure because of its simple implantation process, wide range of sizes, and excellent occlusion rate. However, the original stiffer delivery wire of the ASO transferred considerable tension to the occluder and the atrial septum increasing the risks of device embolization and procedure failure especially in patients with suboptimal atrial morphologies (13, 14). A significant proportion of defects are neither central nor small, and these are more of a challenge for percutaneous closure. Deficiencies of the anterosuperior septum are common, especially in large defects. The common approach from the inferior caval vein means that the ASO will approach the atrial septum at an angle. It can be challenging to prevent the anterosuperior rim of the device from pulling from the left atrium into the right atrium before the core of the device can be developed. Technical modifications to tackle difficult anatomies were reported (11, 12). Modified deployment maneuvers included changing the orientation of the left atrial disk of the device within the left atrium or altering the deployment sequence by delivering the central core of the device slightly within the left atrium before bridging the device back to the septum, and in case of failure to deliver the left disk within the left or right upper lobe pulmonary vein. Modifications in implantation techniques also included the use of modified or steerable delivery sheaths and balloon-assisted closures (15–17). However, all these maneuvers were associated with longer procedures, higher radiation, and an increased risk of complications (12, 14). Therefore, new titanium-coated devices were developed similar to the ASO but with modified delivery systems to overcome the aforementioned difficulties. The second-generation flex of Occlutech Figulla® septal occluder (Occlutech GmbH, Jena, Germany) was introduced in 2009 with improved delivery features (18, 19). The screw mechanism of the ASO was replaced by a ball-shaped connector design that allows a tilt angle up to 45° with better septum alignment during implantation. The system was even upgraded in the third-generation device (Flex II) to a bioptome-like innovative delivery system that comes along with a steerable catheter, allowing full circular movement of the device on the delivery wire and facilitating closure of larger or asymmetric defects (20, 21). However, this device carries 2 major drawbacks (7, 8). Difficulty in selecting the correct device size was reported as the device exists in 3 mm increments for sizes between 21 and 40 mm (7). The Occlutech device is manufactured using thicker diameter Nitinol wire as compared to ASO, requiring larger venous delivery sheaths for a device of equivalent size to the ASO. This is not a problem in adults but its use in children might be limited because of the risk of vascular damage (8). More recently, the CeraFlex^TM^ ASD occluder (Lifetech Scientific, Shenzhen, China) was commercialized as an alternative device to the ASO with structural innovations including a pre-mounted delivery system with 360° flexible connection and minimized amount of implant material (22, 23). Astarcioglu et al. demonstrated comparable outcomes with the ASO with no procedural complications (23). He reported that the operator can evaluate the device's final position without tension from the delivery catheter, allowing safe placement and detachment with the lock/release mechanism. Nevertheless, reported clinical experience with CeraFlex^TM^ ASD occluder is very limited with lacking long-term data about its safety (22–24). The disadvantages of this device also include limited size options (19 vs. 27 sizes in ASO), and the requirement of a slightly larger delivery sheath than with ASO devices. A recent prospective concurrent head-to-head comparison of ASO with Ceraflex and Occlutech ASD occluders showed that the newly-modified structural designs do not show any advantages in terms of procedural complications and midterm follow-up outcomes (10). Georgiev et al. even showed that retrieval of Ceraflex and Occlutech ASD occluders larger than 16 mm was impossible *in vivo* and during benchside using standard snare techniques (9). The authors had to develop and test a new removal method using biopsy forceps which proved to be feasible in animal experiments. In contrast with the ASO, these technical difficulties in percutaneous device removal may be an important drawback for clinical application. On the other hand, always keep in mind that despite the great success of the ASO, the risk of erosion remains a safety caution, especially since it can be fatal and may occur several years after implantation (25). However, device erosion was also reported with many other newer devices (26, 27). It is here where the Carag Bioresorbable Septal Occluder (Carag AG, Baar, Switzerland) stands out here as a breakthrough novel device that advances septal occluder technology to a new level by eliminating the risks of perforation and valve distortion. Yet, no detailed technical data and clinical feedback have been published and only 3 sizes are available for defects up to 25 mm (25, 28).

**Figure 5:**
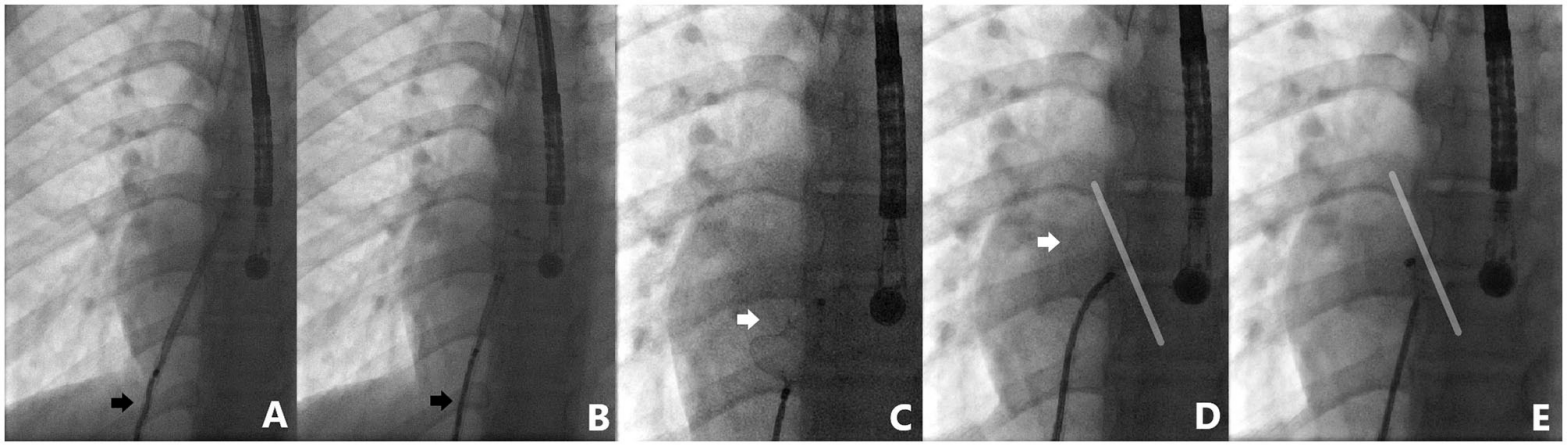
Case 6 presentation. Detailed fluoroscopic views of device deployment sequence. Note how the cable adapts to the degree of applied tension during left disk deployment (black arrows) **(A,B)**. Minnesota wiggle maneuver performed with complete confidence **(C,D)**. Identical device position before and after release **(D,E)**.

The ATIDS was designed and developed by the Amplatzer manufacturers to deliver their devices with promises of increased procedural flexibility and deployment control. Our patients experienced successful implantations with no major complications during or after the procedure. The delivery system handled very well as device movement across the sheath was not affected by the new applied technologies. Based on our preliminary experience, this new design will enable interventionists to perform classic and complex ASD closures with complete ease and confidence. The recently added black marking on the cable enabled blind delivery of the device until the tip of the sheath, theoretically reducing fluoroscopic time. We noticed that the device was perfectly aligned with the septum in all cases (Figures 2–5). When the ASD had to be closed using the transjugular approach, ATIDS offered the major advantage of positioning the device in the plane of the septum without the use of a steerable introducer. The perpendicular orientation of the delivery system to the atrial septum was easily obtained with the ultra-flexible wire (Figures 3B,C). The flexible 3-dimensional flexibility with a tilt angle going up to 120° (Figures 4C,D) helped in conforming the ASO to the various septal anatomies, minimizing maneuvers, and possibly the post-occlusion para-prosthetic leaks. The increased flexibility on the wire tip decreased the tension created by the cable on the device and subsequently on the atrial septum. This reduced tension allowed a more controlled deployment sequence helping us in positioning the device more easily into the desired position and minimizing any unwanted drag or pull on the implant. However, the ability to retrieve and redeploy the disks for proper positioning was identical to the original delivery wire (previous center experience), allowing appropriate repositioning when necessary. The secure and stable device position within the defect can also be checked by the “Minnesota wiggle” (Figures 5C,D). The sheath only needs to be advanced toward the occluder to stabilize the ultra-flexible section of the cable and allow the push-pull manoeuver with complete security and confidence. Most importantly, the ATIDS, in comparison to the classic delivery system offered the major advantage of accurate assessment of device final position before cable release and therefore a more reliable TOE as well as TEE assessment of the device accommodation over the margins and the presence of a residual para-prosthetic leak (Figure 2). When the device is deployed into position, we recommend a clockwise rotation of the delivery sheath and a gentle push on the cable until the ultra-flexible wire-tip curves into a 45° to 90° angle (Figures 2B,C). These maneuvers will enable interventionists to benefit from the maximum flexibility of the Trevisio™ cable, eliminating the tension on the device that will conform to its final and definitive position (Figures 2D,G). The shape, position, and orientation of the device were strictly identical before and after release in all cases (Figures 2, 3, 5). The forecasting of device configuration before cable release will certainly help in improving procedural outcomes, especially in complex and unclassical cases.

## Conclusion

The safety and efficacy of the newly developed ATIDS were described in our case-series of pediatric ASD transcatheter closures. The increased implantation flexibility and reduced tension during ASO positioning were remarkable as the definitive device position on the atrial septal wall was observed before cable release minimizing any unwanted drag or pull on the implant and improving procedure outcomes. With the enhanced maneuverability and flexibility, ATIDS will probably be beneficial in other types of percutaneous procedures such as ventricular septal defect and patent arterial duct closures.

## Data Availability Statement

The raw data supporting the conclusions of this article will be made available by the authors, without undue reservation.

## Ethics Statement

The authors assert that all procedures contributing to this work comply with the ethical standards of the relevant national guidelines on human experimentation, and with the Helsinki Declaration of 1975, as revised in 2008. Approval from the institutional review board was obtained (MR004: 2020-1117150323). Written informed consent to participate in this study was provided by the participants' legal guardian/next of kin.

## Author Contributions

RH collected and critically analyzed all clinical data, designed the study and all illustrative material, and took the lead in writing the entire manuscript. All authors discussed the results, read, and approved the final manuscript.

## Conflict of Interest

SM-M received fundings from Abbott to travel to scientific meetings. The remaining authors declare that the research was conducted in the absence of any commercial or financial relationships that could be construed as a potential conflict of interest.

## References

[1] King TD Thompson SL Steiner C Mills NL. Secundum atrial septal defect. Nonoperative closure during cardiac catheterization. JAMA. (1976) 235:2506–9. 10.1001/jama.235.23.2506 946659

[2] Rickers C Hamm C Stern H Hofmann T Franzen O Schräder R. Percutaneous closure of secundum atrial septal defect with a new self centering device (“angel wings”). Heart. (1998) 80:517–21. 10.1136/hrt.80.5.517 9930056 PMC1728848

[3] Sharafuddin MJ Gu X Titus JL Urness M Cervera-Ceballos JJ Amplatz K. Transvenous closure of secundum atrial septal defects: preliminary results with a new self-expanding nitinol prosthesis in a swine model. Circulation. (1997) 95:2162–8. 10.1161/01.CIR.95.8.2162 9133527

[4] Masura J Gavora P Formanek A Hijazi ZM. Transcatheter closure of secundum atrial septal defects using the new self-centering amplatzer septal occluder: initial human experience. Cathet Cardiovasc Diagn. (1997) 42:388–93. 10.1002/(SICI)1097-0304(199712)42:4<388::AID-CCD7>3.0.CO;2-7 9408617

[5] Du ZD Hijazi ZM Kleinman CS Silverman NH Larntz K Amplatzer Investigators. Comparison between transcatheter and surgical closure of secundum atrial septal defect in children and adults: results of a multicenter nonrandomized trial. J Am Coll Cardiol. (2002) 39:1836–44. 10.1016/S0735-1097(02)01862-4 12039500

[6] Berger F Vogel M Alexi-Meskishvili V Lange PE. Comparison of results and complications of surgical and Amplatzer device closure of atrial septal defects. J Thorac Cardiovasc Surg. (1999) 118:674–8; discussion 678-80. 10.1016/S0022-5223(99)70013-9 10504632

[7] Pac A Polat TB Cetin I Oflaz MB Balli S. Figulla ASD occluder versus Amplatzer Septal Occluder: a comparative study on validation of a novel device for percutaneous closure of atrial septal defects. J Interv Cardiol. (2009) 22:489–95. 10.1111/j.1540-8183.2009.00497.x 19735475

[8] Godart F Houeijeh A Recher M Francart C Polge AS Richardson M. Transcatheter closure of atrial septal defect with the Figulla(®) ASD Occluder: a comparative study with the Amplatzer(®) Septal Occluder. Arch Cardiovasc Dis. (2015) 108:57–63. 10.1016/j.acvd.2014.09.005 25453168

[9] Georgiev S Tanase D Genz T Ewert P Naumann S Pozza RD. Retrieval of large Occlutech Figula Flex septal defect occluders using a commercially available bioptome: proof of concept. Cardiol Young. (2018) 28:955–60. 10.1017/S1047951118000586 29779498

[10] Bhattacharjya S Pillai LS Doraiswamy V Satyanarayana RM Chandrasekaran R Pavithran S. Prospective concurrent head-to head comparison of three different types of nitinol occluder device for transcatheter closure of secundum atrial septal defects. EuroIntervention. (2019) 15:e321–8. 10.4244/EIJ-D-18-01016 30946015

[11] Haddad RN Maleux G Bonnet D Malekzadeh-Milani S. Transhepatic atrial septal defect closure: simple way to achieve haemostasis in a patient with important co-morbidities. Cardiol Young. (2020) 30:1343–5. 10.1017/S1047951120001833 32635957

[12] Haddad RN Abdel Massih T Saliba Z. Not just another large atrial septal defect: complex anatomy, challenging procedure, and an unusual complication. Cardiol Young. (2020) 30:1052–6. 10.1017/S1047951120001468 32539899

[13] Fraisse A Latchman M Sharma SR Bayburt S Amedro P di Salvo G. Atrial septal defect closure: indications and contra-indications. J Thorac Dis. (2018) 10(Suppl. 24):S2874–81. 10.21037/jtd.2018.08.111 30305947 PMC6174144

[14] Faccini A Butera G. Atrial septal defect (ASD) device trans-catheter closure: limitations. J Thorac Dis. (2018) 10(Suppl. 24):S2923–30. 10.21037/jtd.2018.07.128 30305952 PMC6174146

[15] Spies C Boosfeld C Schräder R. A modified Cook sheath for closure of a large secundum atrial septal defect. Catheter Cardiovasc Interv. (2007) 70:286–9. 10.1002/ccd.21082 17630671

[16] Nounou M Harrison A Kern M. A novel technique using a steerable guide catheter to successfully deliver an Amplatzer septal occluder to close an atrial septal defect. Catheter Cardiovasc Interv. (2008) 72:994–7. 10.1002/ccd.21743 19021288

[17] Narin N Baykan A Argun M Ozyurt A Pamukcu O Bayram A. New modified balloon-assisted technique to provide appropriate deployment in the closure of large secundum atrial septal defect using amplatzer septal occluder in children. J Invasive Cardiol. (2014) 26:597–602. 10.1016/S0167-5273(13)70026-3 25364001

[18] Roymanee S Promphan W Tonklang N Wongwaitaweewong K. Comparison of the Occlutech® Figulla® septal occluder and Amplatzer® septal occluder for atrial septal defect device closure. Pediatr Cardiol. (2015) 36:935–41. 10.1007/s00246-015-1103-y 25633819

[19] Haas NA Soetemann DB Ates I Baspinar O Ditkivskyy I Duke C. Closure of secundum atrial septal defects by using the occlutech occluder devices in more than 1300 patients: the IRFACODE project: a retrospective case series. Catheter Cardiovasc Interv. (2016) 88:571–81. 10.1002/ccd.26497 27029396

[20] Snijder RJR Renes LE Bosshardt D Suttorp MJ Ten Berg JM Post MC. Percutaneous atrial septal defect closure using the occlutech figulla device in adults: more than 800 patient-years of follow-up. J Interv Cardiol. (2020) 2020:7136802. 10.1155/2020/7136802 32140088 PMC7042503

[21] Kenny D Eicken A Dähnert I Boudjemline Y Sievert H Schneider MB. A randomized, controlled, multi-center trial of the efficacy and safety of the Occlutech Figulla Flex-II Occluder compared to the Amplatzer Septal Occluder for transcatheter closure of secundum atrial septal defects. Catheter Cardiovasc Interv. (2019) 93:316–21. 10.1002/ccd.27899 30719850

[22] Apostolopoulou SC Tsoutsinos A Laskari C Kiaffas M Rammos S. Large single centre experience with the Cera™ and CeraFlex™ occluders for closure of interatrial communications: usefulness of the flexible rotation feature. Cardiovasc Interv Ther. (2018) 33:70–6. 10.1007/s12928-016-0440-y 27832479

[23] Astarcioglu MA Kalcik M Sen T Aykan AC Gokdeniz T Gursoy OM. Ceraflex versus Amplatzer occluder for secundum atrial septal defect closure. Multicenter clinical experience. Herz. (2015) 40(Suppl. 2):146–50. 10.1007/s00059-014-4192-0 25662695

[24] Yücel IK Balli S Küçük M Çelebi A. Use of steerable delivery catheter to successfully deliver a Ceraflex septal occluder to close an atrial septal defect in a child with interrupted inferior vena cava with azygos continuation. Turk Kardiyol Dern Ars. (2016) 44:244–7. 10.5543/tkda.2015.75222 27138315

[25] Nassif M Abdelghani M Bouma BJ Straver B Blom NA Koch KT. Historical developments of atrial septal defect closure devices: what we learn from the past. Expert Rev Med Devices. (2016) 13:555–68. 10.1080/17434440.2016.1182860 27112301

[26] Auriau J Bouvaist H Aaberge L Abe T Dähnert I Panzer J. Cardiac erosions after transcatheter atrial septal defect closure with the occlutech figulla flex device. JACC Cardiovasc Interv. (2019) 12:1397–9. 10.1016/j.jcin.2019.03.005 31320034

[27] Kumar P Orford JL Tobis JM. Two cases of pericardial tamponade due to nitinol wire fracture of a gore septal occluder. Catheter Cardiovasc Interv. (2020) 96:219–24. 10.1002/ccd.28596 31696617

[28] Sigler M Söderberg B Schmitt B Mellmann A Bernhard J. Carag bioresorbable septal occluder (CBSO): histopathology of experimental implants. EuroIntervention. (2018) 13:1655–61. 10.4244/EIJ-D-17-00006 28555594

